# Increase in Travel-Associated and Locally Acquired Dengue Cases — United States, 2024

**DOI:** 10.15585/mmwr.mm7518a1

**Published:** 2026-05-14

**Authors:** Sandra J. Kiplagat, Dania M. Rodriguez, Aidsa Rivera, Gabriela Paz-Bailey, Joshua M. Wong, Laura E. Adams

**Affiliations:** ^1^Epidemic Intelligence Service, CDC; ^2^Dengue Branch, Division of Vector-Borne Diseases, National Center for Emerging and Zoonotic Infectious Diseases, CDC, San Juan, Puerto Rico.

**Summary**

**What is already known about this topic?**

Dengue is a leading cause of febrile illness in travelers returning from regions where the virus is endemic.

**What is added by this report?**

During 2024, the number of dengue cases reported from U.S. states and the District of Columbia increased 359% above the annual average during 2010–2023; a total of 97.2% of cases were travel associated, and 2.8% were locally acquired. Approximately one fifth (21.8%) of cases occurred among persons aged 50–59 years, and more than one half (57.5%) occurred in Hispanic or Latino persons. Approximately one third (36.1%) of patients were hospitalized; six (0.2%) patients died.

**What are the implications for public health practice?**

The sharp increase in travel-associated dengue highlights an urgent need for enhanced prevention strategies, improved clinical awareness, and tailored messaging for travelers to areas with ongoing dengue transmission. 

## Abstract

Dengue is a mosquitoborne viral disease that can cause mild to severe illness and death. During 2010–2023, an average of 828 dengue cases (range = 202–2,055) were reported annually to ArboNET, the national arboviral surveillance system for the 50 U.S. states and the District of Columbia. During 2024, a record 3,798 dengue cases were reported, representing a 359% increase above the 2010–2023 annual average. Among these 3,798 cases, 97.2% were associated with travel outside the reporting jurisdiction during the 2 weeks preceding symptom onset; the remaining 2.8% were locally acquired. The number of dengue cases peaked during July–September (accounting for 41.6% of total annual cases), with the highest percentage (21.8%) of cases occurring among persons aged 50–59 years. Among travel-associated cases, acquisition occurred primarily in the Caribbean (including Puerto Rico and the U.S. Virgin Islands) (34.1%), North America (Mexico and the United States) (24.3%), and Central America (15.6%); Hispanic or Latino persons accounted for 57.5% of all cases. Among all patients, 36.1% required hospitalization, 2.8% of cases were severe, and six (0.2%) patients died. Among 1,204 patients with known dengue virus (DENV) serotype, DENV-3 was the most commonly reported (54.8%) among the four DENV serotypes. These findings underscore the urgent need for enhanced prevention strategies, clinical awareness, and tailored public health messaging for travelers to areas where dengue is endemic.

## Introduction

Dengue, a mosquitoborne viral disease caused by four distinct dengue virus (DENV) types (1–4), is a leading cause of febrile illness in travelers returning to the United States from regions where the virus is endemic (*1*). Illness can be mild or life-threatening (*2*). Frequent or continuous DENV transmission in the United States is limited to six U.S. territories and freely associated states*; however, dengue cases occur annually among residents of the 50 U.S. states and the District of Columbia (DC) who travel to areas where dengue is endemic, and sporadic locally acquired cases have been reported from limited areas of the continental United States with competent mosquito vectors. During 2010–2023, an average of 828 dengue cases (range = 202–2,055) among residents of the 50 U.S. states and DC were reported annually to ArboNET, the national arboviral surveillance system; the majority of cases were travel associated. During 2024, a sharp increase in cases was reported among travelers returning from areas with endemic dengue (*3*), highlighting the importance of identifying and evaluating travel-associated cases, which might result in higher risk for autochthonous transmission in parts of the United States where dengue is not endemic. To better understand travel-associated and locally acquired dengue, this analysis describes dengue cases reported to CDC during 2024 from the 50 U.S. states and DC.

## Methods

### Data Source

Confirmed and probable dengue cases^†^ reported to CDC from U.S. states and DC among persons with symptom onset in 2024 were included in the analysis. Cases were classified as dengue, denguelike illness, or severe dengue using the 2015 Council of State and Territorial Epidemiologists case definitions.^§^ Case numbers are preliminary and might not reflect final counts for 2024.

### Analysis

Travel-associated cases were defined as those that occurred in persons who traveled outside their reporting jurisdiction in the 2 weeks preceding symptom onset. Persons who did not travel in the 2 weeks preceding symptom onset were considered to have locally acquired infections. The characteristics analyzed included case status, sex, age group, race and ethnicity, month of infection, patient outcome, DENV serotype, and most likely location of exposure. Incidences were calculated using 2024 U.S. Census Bureau population estimates. R (version 4.5.0; R Core Team) was used to conduct all analyses. This activity was reviewed by CDC, deemed not research, and conducted consistent with applicable federal law and CDC policy.^¶^

## Results

### Travel-Associated and Locally Acquired Dengue Cases

During 2024, a total of 3,798 dengue cases, including 2,252 (59.3%) confirmed and 1,546 (40.7%) probable cases, were reported to CDC from the 50 U.S. states and DC. These represent a 359% increase above the annual average of 828 cases per year during 2010–2023. Among these cases, 3,693 (97.2%) were travel associated, and 105 (2.8%) were locally acquired (Table 1). Overall, 1,581 (41.6%) of all travel associated and locally acquired cases occurred during the summer months, including 509 (13.4%) in July, 602 (15.9%) in August, and 470 (12.4%) in September (Figure).

**TABLE 1:** Characteristics of confirmed and probable travel-associated and locally acquired dengue cases — National Arbovirus Surveillance System, 50 U.S. states and the District of Columbia, 2024

Characteristic	No. (%)		
	Travel associated	Locally acquired	Total
**Total**	**3,693 (97.2)**	**105 (2.8)**	**3,798 (100.0)**
Case status*			
Confirmed	2,165 (58.6)	87 (82.9)	**2,252 (59.3)**
Probable	1,528 (41.4)	18 (17.1)	**1,546 (40.7)**
**Sex**			
Female	1,946 (52.7)	49 (46.7)	**1,995 (52.5)**
Male	1,745 (47.3)	56 (53.3)	**1,801 (47.4)**
Unknown	2 (0.1)	—	**2 (0.1)**
**Age group, yrs, median (IQR)**	49 (31–61)	46 (31–57)	**49 (31–61)**
0–10	110 (3.0)	2 (1.9)	**112 (2.9)**
11–19	383 (10.4)	10 (9.5)	**393 (10.3)**
20–29	397 (10.8)	12 (11.4)	**409 (10.8)**
30–39	481 (13.0)	13 (12.4)	**494 (13.0)**
40–49	582 (15.8)	25 (23.8)	**607 (16.0)**
50–59	806 (21.8)	23 (21.9)	**829 (21.8)**
60–69	570 (15.4)	8 (7.6)	**578 (15.2)**
≥70	364 (9.9)	12 (11.4)	**376 (9.9)**
**Race and ethnicity **			
Hispanic or Latino (any race)	2,115 (57.3)	70 (66.7)	**2,185 (57.5)**
American Indian or Alaska Native, NH	3 (0.1)	—	** 3 (0.1)**
Asian, NH	229 (6.2)	3 (2.9)	**232 (6.1)**
Black or African American, NH	95 (2.6)	—	**95 (2.5)**
Native Hawaiian or Pacific Islander, NH	10 (0.3)	—	**10 (0.3)**
White, NH	605 (16.4)	28 (26.7)	**633 (16.7)**
Other/Multiple races	154 (4.2)	1 (1.0)	**155 (4.1)**
Unknown race and ethnicity	482 (13.1)	3 (2.9)	**485 (12.8)**
**Month of illness onset **			
Jan–Mar	636 (17.2)	6 (5.7)	**642 (16.9)**
Apr–Jun	594 (16.1)	10 (9.5)	**604 (15.9)**
Jul–Sep	1,528 (41.4)	53 (50.5)	**1,581 (41.6)**
Oct–Dec	935 (25.3)	36 (34.3)	**971 (25.6)**
**Dengue classification^†^**			
Denguelike illness	51 (1.4)	3 (2.9)	**54 (1.4)**
Dengue	3,538 (95.8)	101 (96.2)	**3,639 (95.8)**
Severe dengue	104 (2.8)	1 (1.0)	**105 (2.8)**
**Hospitalized **			
Yes	1,321 (35.8)	49 (46.7)	**1,370 (36.1)**
No	2,271 (61.5)	56 (53.3)	**2,327 (61.3)**
Unknown	101 (2.7)	—	**101 (2.7)**
**Outcome **			
Survived	3,522 (95.4)	104 (99.0)	**3,626 (95.5)**
Died	6 (0.2)	—	**6 (0.2)**
Unknown	165 (4.5)	1 (1.0)	**166 (4.4)**
**DENV serotype (among 1,204 cases with known serotype)**			
DENV-1	142 (12.5)	9 (13.0)	**151 (12.5)**
DENV-2	114 (10.0)	—	**114 (9.5)**
DENV-3	607 (53.5)	53 (76.8)	**660 (54.8)**
DENV-4	272 (24.0)	7 (10.1)	**279 (23.2)**
**Location of exposure **			
International (outside of U.S. states or territories)	3,259 (88.2)	—	**3,259 (85.8)**
Within a U.S. state or territory	246 (6.7)	105 (100.0)	**351 (9.2)**
Unknown	188 (5.1)	—	**188 (4.9)**
**U.S. state or territory of acquisition of travel-associated dengue (n = 246)**			
Puerto Rico	188 (76.4)	—	**188 (76.4)**
U.S. Virgin Islands	54 (22.0)	—	**54 (22.0)**
Florida	4 (1.6)	—	**4 (1.6)**
**Region of travel acquisition **			
Caribbean^§^	1,259 (34.1)	—	**1,259 (34.1)**
North America	896 (24.3)	—	**896 (24.3)**
Central America	577 (15.6)	—	**577 (15.6)**
Asia	373 (10.1)	—	**373 (10.1)**
South America	346 (9.4)	—	**346 (9.4)**
Africa	40 (1.1)	—	**40 (1.1)**
Oceania	11 (0.3)	—	**11 (0.3)**
Europe	2 (0.1)	—	**2 (0.1)**
Multiple regions^¶^	1 (0)	—	**1 (0)**
Unknown	188 (5.1)	—	**188 (5.1)**

**Abbreviations**: DENV = dengue virus; IgM = immunoglobulin M; NH = non-Hispanic.

* Confirmed cases required detection of DENV RNA by reverse transcription–polymerase chain reaction, DENV antigen, nonstructural protein 1 antigen, or IgM anti-DENV antibody without likely flavivirus cross exposure; probable cases had anti-DENV IgM detected among persons living in or traveling to an area with evidence of other flavivirus transmission. Dengue 2015 Case Definition | CDC

^†^ Dengue is defined as the presence of fever (as reported by the patient or a health care provider) and one or more of the following signs and symptoms: nausea or vomiting, rash, aches and pains (e.g., headache, retro-orbital pain, joint pain, myalgia, or arthralgia), positive tourniquet test, leukopenia (total white blood cell count <5,000/mm^3^), or any warning sign for severe dengue (i.e., abdominal pain or tenderness, persistent vomiting, or extravascular fluid accumulation [e.g., pleural or pericardial effusion or ascites]), mucosal bleeding at any site, liver enlargement of >0.8 in (>2 cm), or increasing hematocrit concurrent with a rapid decrease in platelet count. Denguelike illness is defined as the presence of fever as reported by the patient or a health care provider. Severe dengue is defined as dengue with any one or more of the following: 1) severe plasma leakage evidenced by hypovolemic shock or extravascular fluid accumulation with respiratory distress; 2) severe bleeding from the gastrointestinal tract or vagina requiring medical intervention; or 3) severe organ involvement, including elevated liver transaminases (aspartate aminotransferase or alanine aminotransferase ≥1,000 U/L), impaired consciousness, or heart or other organ involvement.

^§^ Includes all Caribbean islands, including Puerto Rico and the U.S. Virgin Islands.

^¶^ Includes persons who traveled to more than one region.

**FIGURE:**
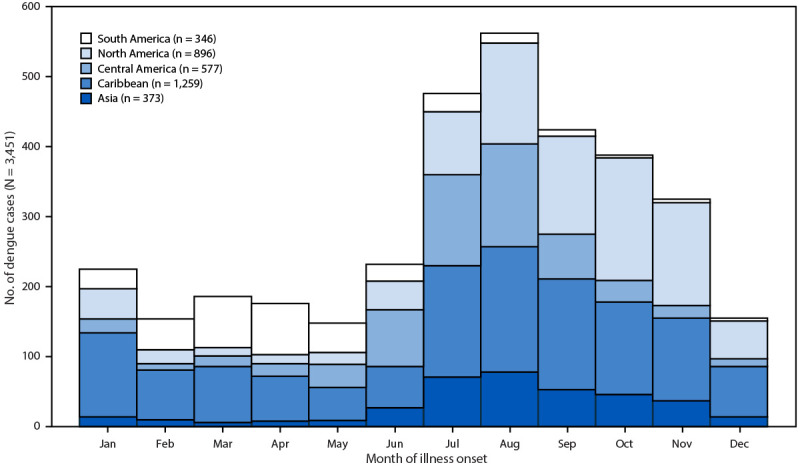
Travel-associated dengue cases, by region* of travel and month of illness onset — National Arbovirus Surveillance System, United States, 2024 * Includes regions with 50 or more cases. A total of 242 travel-associated cases are not included for Africa (40 cases), Oceania (11), Europe (two), multiple regions (one), and unknown locations (188). North America includes Mexico and the United States. The Caribbean and Central America are presented separately.

### Characteristics of Patients with Dengue

The median patient age was 49 years (IQR = 31–61 years); approximately one fifth of cases (21.8%) occurred in adults aged 50–59 years, followed by 16% in adults aged 40–49 years. Females accounted for 52.5% of cases. Overall, 2,185 (57.5%) patients identified as Hispanic or Latino (Hispanic), as did two thirds (66.7%) of patients with locally acquired cases.

Among all patients with travel-associated or locally acquired dengue, 54 (1.4%) had denguelike illness, 3,639 (95.8%) had dengue, and 105 (2.8%) had severe dengue. More than one third (36.1%) of patients required hospitalization. Six (0.2%) deaths were reported, all among patients with travel-associated DENV infection who developed severe dengue. The highest case-fatality rate (CFR) (0.36%) occurred among adults aged 50–59 years; three deaths occurred among 829 patients in this age group, followed by two deaths among 578 adults aged 60–69 years (CFR = 0.35%), and one death among 409 adults aged 20–29 years (CFR = 0.24%).

### DENV Serotypes

DENV serotype was available for 1,204 (31.7%) dengue cases. Among these, DENV-3 was the most commonly identified serotype (54.8%), followed by DENV-4 (23.2%), DENV-1 (12.5%), and DENV-2 (9.5%).

### Geographic Distribution of Cases

The highest number of dengue cases among residents of U.S. states and DC occurred in Florida (1,044; 27.5% of cases), followed by California (720; 19.0%), New York (338; 8.9%), and Texas (241; 6.3%) (Table 2). The highest dengue incidence (4.47 per 100,000 population) occurred in Florida and was approximately four times the national incidence (1.12). Three jurisdictions reported locally acquired cases, including Florida (85 cases reported from 10 counties), California (18 from three counties), and Texas (two from two counties).

**TABLE 2:** Travel-associated and locally acquired dengue cases, by state — National Arbovirus Surveillance System, United States, 2024

Jurisdiction	Total no. of cases (column %)	No. (row %)		Incidence*
		Locally acquired	Travel associated	
**United States**	**3,798 (100.0)**	**105 (2.8)**	**3,693 (97.2)**	**1.12**
Florida	**1044**	85	959	4.47
California	**720**	18	702	1.83
New York	**338**	0	338	1.70
Texas	**241**	2	239	0.77
Massachusetts	**143**	0	143	2.00
New Jersey	**127**	0	127	1.34
Illinois	**119**	0	119	0.94
Maryland	**80**	0	80	1.28
North Carolina	**70**	0	70	0.63
Washington	**67**	0	67	0.84
Pennsylvania	**66**	0	66	0.50
Georgia	**56**	0	56	0.50
Arizona	**53**	0	53	0.70
Colorado	**48**	0	48	0.81
Virginia	**47**	0	47	0.53
Connecticut	**45**	0	45	1.22
Minnesota	**44**	0	44	0.76
Michigan	**43**	0	43	0.42
Wisconsin	**39**	0	39	0.65
Oregon	**34**	0	34	0.80
Ohio	**33**	0	33	0.28
Indiana	**27**	0	27	0.39
Utah	**25**	0	25	0.71
Louisiana	**25**	0	25	0.54
South Carolina	**25**	0	25	0.46
Rhode Island	**19**	0	19	1.71
Tennessee	**19**	0	19	0.26
Hawaii	**16**	0	16	1.11
Kentucky	**15**	0	15	0.33
Arkansas	**14**	0	14	0.45
Nevada	**14**	0	14	0.43
Vermont	**13**	0	13	2.00
District of Columbia	**13**	0	13	1.85
New Mexico	**13**	0	13	0.61
Missouri	**12**	0	12	0.19
Alabama	**11**	0	11	0.21
New Hampshire	**10**	0	10	0.71
Nebraska	**10**	0	10	0.50
Iowa	**9**	0	9	0.28
Kansas	**8**	0	8	0.27
Oklahoma	**8**	0	8	0.20
Delaware	**7**	0	7	0.67
Idaho	**7**	0	7	0.35
Maine	**5**	0	5	0.36
Mississippi	**4**	0	4	0.14
Alaska	**3**	0	3	0.41
South Dakota	**3**	0	3	0.32
Montana	**3**	0	3	0.26
West Virginia	**2**	0	2	0.11
Wyoming	**1**	0	1	0.17

* Cases per 100,000 population, calculated using data from the U.S. Census Bureau.

### Reported Travel Among Persons with Travel-Associated Dengue

Among the 3,693 persons with travel-associated dengue, 3,259 (88.2%) reported international travel (outside U.S. states and territories), and 246 (6.7%) reported travel to a U.S. state or territory. Most of the 246 cases among travelers to U.S. jurisdictions occurred in Puerto Rico (76.4%) or the U.S. Virgin Islands (22.0%); 1.6% occurred in Florida. Among the 3,505 (94.9%) persons with travel-associated dengue and known exposure location, the most frequent destinations were Mexico (892; 25.4%), Cuba (633; 18.1%), and India (225; 6.4%) (Supplementary Figure). Among all travel-associated cases, the most frequently visited regions were the Caribbean (including Puerto Rico and the U.S. Virgin Islands [34.1%]), North America (Mexico and the United States) (24.3%), Central America (15.6%) and Asia (10.1%).** Trends varied by month, with more cases occurring in persons returning from South America during January–May and more in persons returning from the Caribbean, North America, Central America, and Asia during July–November (Figure).

## Discussion

The record number of dengue cases reported in residents of U.S. states and DC in 2024 represented a 359% increase above the annual average of 828 reported cases during 2010–2023. The 2024 increase was primarily caused by an increase in travel-associated cases (97.2% of reported dengue cases) and reflects the sharp global surge in dengue cases observed in 2024 (*3*). Worldwide, dengue cases reached 14.1 million in 2024, including approximately 13 million cases in the region of the Americas, an increase of approximately 8 million cases compared with 2023 (*3*,*4*). In U.S. states, cases increased seasonally among residents who traveled to areas experiencing dengue outbreaks, with more cases among travelers to South America during the first half of the year and more cases among travelers to Central America and the Caribbean during the second half of the year (*3*). Dengue cases among residents of the continental United States who traveled to the Caribbean included those in persons who traveled to the U.S. territories of Puerto Rico and the U.S. Virgin Islands; both territories reported dengue outbreaks in 2024 (*5*).

Locally acquired cases in Florida, California, and Texas were reported from multiple counties within each state, suggesting multiple introduction events rather than extensive ongoing transmission in one location. Although these introductions did not lead to larger outbreaks in the continental United States, they indicate an increasing risk for locally acquired cases, underscoring the need for enhanced surveillance, vector control, and public health preparedness and response efforts in areas with competent mosquito vectors. *Aedes aegypti* and *Aedes albopictus* mosquitoes are present in many counties in both the eastern and western United States and pose an ongoing risk for DENV transmission in new areas. Approximately one half of U.S. counties have competent mosquito vectors, and the climate in three quarters of the United States is suitable for *Aedes* species mosquitoes (*6*). Vector presence and distribution might be further affected by increasing temperatures, which can expand the range of mosquitoes that spread dengue, enhance vector survival, and change the reproduction and biting rates (*7*).

A majority of dengue cases occurred among patients reporting Hispanic ethnicity. This finding likely reflects travel patterns, because Hispanic persons might travel more frequently to areas with endemic dengue, such as Latin America and the Caribbean; these areas experienced large dengue outbreaks in 2024 (*8*,*9*). Approximately one third of travelers with dengue were hospitalized. In addition, 105 patients met the criteria for severe dengue, which can be associated with life-threatening complications, including severe bleeding, plasma leakage, and organ impairment; 92 (88%) patients with severe dengue were hospitalized. Although cases and hospitalizations occurred among all age groups, CFR was highest among adults aged 50–59 years, followed by those aged 60–69 years. This pattern might reflect the higher prevalence of underlying medical conditions and increased risk for severe outcomes among older adults.

### Limitations

The findings in this report are subject to at least three limitations. First, reported case counts are an underestimate of the true number of infections, because many persons with dengue have mild symptoms or might not seek health care services. Second, dengue diagnoses might be missed if health care providers do not suspect dengue or test accordingly, also resulting in an underestimate of the number of infections. Finally, underreporting might vary by jurisdiction because of differences in dengue awareness, surveillance capacity, and public health infrastructure.

### Implications for Public Health Practice

A record-high number of dengue cases, primarily travel associated, were reported among residents of U.S. states and DC in 2024, highlighting an urgent need for coordinated dengue prevention and response efforts across public health agencies, clinical settings, and vector control programs. Public health and clinical partners can use the CDC Yellow Book and Travel Health Notices to strengthen messaging to U.S. travelers about places where dengue outbreaks are occurring and recommended protective measures such as Environmental Protection Agency (EPA)–approved repellents; wearing loose-fitting long-sleeved pants and shirts; and using air conditioning and window screens when visiting regions with endemic dengue. Public health authorities can also play an essential role in conducting case investigations, reporting dengue cases, and educating communities at risk for dengue about prevention strategies. Given the disproportionate impact of dengue on Hispanic communities and travelers, culturally tailored public health messaging might help mitigate travel-related risk, particularly among persons visiting regions with endemic dengue in Latin America and the Caribbean. Clinicians should maintain a high index of suspicion for dengue in febrile patients returning from affected areas and be prepared to order appropriate diagnostic testing and effectively manage cases, including monitoring patients for warning signs that might indicate progression to severe dengue, such as severe abdominal pain, persistent vomiting, mucosal bleeding, or altered mental status (Dengue Clinical Management Pocket Guide | Dengue | CDC).

Although no dengue vaccines are currently approved for U.S. travelers who are visiting but not living in dengue-endemic areas (*10*), persons can protect themselves by preventing mosquito bites during travel to and after returning from areas with dengue risk where competent mosquito vectors are present to reduce the risk and potential for local transmission. Recommended prevention strategies include applying EPA-approved repellents; wearing loose-fitting long-sleeved pants and shirts; using air conditioning and window screens when available; and eliminating standing water containers around homes to prevent mosquitoes from laying eggs.

Dengue is a public health threat to persons of all ages. Enhanced surveillance by vector control programs and implementation of targeted interventions to reduce *A. aegypti* populations in areas with ongoing dengue transmission can mitigate the risk for local establishment of dengue transmission.

## Footnotes

* Frequent or continuous risk is considered to be evidence of more than 10 locally acquired dengue cases in ≥3 of the previous 10 years. Dengue occurs frequently or continuously in the U.S. territories of American Samoa, Puerto Rico, and the U.S. Virgin Islands, and the freely associated states, including the Federated States of Micronesia, Marshall Islands, and Palau. Areas with Risk of Dengue | Dengue | CDC; Data and Statistics on Dengue in the United States | Dengue | CDC

† Confirmed cases required detection of DENV RNA by reverse transcription–polymerase chain reaction, DENV antigen, nonstructural protein 1 antigen, or immunoglobulin M (IgM) anti-DENV antibody without likely flavivirus cross exposure; probable cases had anti-DENV IgM detected among persons living in or traveling to an area with evidence of other flavivirus transmission. Dengue Virus Infections 2015 Case Definition | CDC

§ Dengue is defined as the presence of fever (as reported by the patient or a health care provider) and one or more of the following signs and symptoms: nausea or vomiting, rash, aches and pains (e.g., headache, retro-orbital pain, joint pain, myalgia, or arthralgia), positive tourniquet test, leukopenia (total white blood cell count <5,000/mm^3^), or any warning sign for severe dengue (i.e., abdominal pain or tenderness, persistent vomiting, or extravascular fluid accumulation [e.g., pleural or pericardial effusion or ascites]), mucosal bleeding at any site, liver enlargement of >0.8 in (>2 cm), or increasing hematocrit concurrent with a rapid decrease in platelet count. Denguelike illness is defined as the presence of fever as reported by the patient or a health care provider. Severe dengue is defined as dengue with any one or more of the following: 1) severe plasma leakage evidenced by hypovolemic shock or extravascular fluid accumulation with respiratory distress; 2) severe bleeding from the gastrointestinal tract or vagina requiring medical intervention; or 3) severe organ involvement, including elevated liver transaminases (aspartate aminotransferase or alanine aminotransferase ≥1,000 U/L), impaired consciousness, or heart or other organ involvement.

¶ 45 C.F.R. part 46.102(l)(2), 21 C.F.R. part 56; 42 U.S.C. Sect. 241(d); 5 U.S.C. Sect. 552a; 44 U.S.C. Sect. 3501 et seq.

** Given the high dengue incidence within subregions of North America, three regions were defined for analytic purposes. The Caribbean includes all Caribbean islands, including Puerto Rico and the U.S. Virgin Islands. Central America includes Costa Rica, El Salvador, Guatemala, Honduras, Nicaragua, and Panama. North America includes Mexico and the United States.
